# Ability of radiofrequency echographic multispectrometry to identify osteoporosis status in elderly women with type 2 diabetes

**DOI:** 10.1007/s40520-021-01889-w

**Published:** 2021-05-29

**Authors:** Carla Caffarelli, Maria Dea Tomai Pitinca, Antonella Al Refaie, Elena Ceccarelli, Stefano Gonnelli

**Affiliations:** Department of Medicine, Surgery and Neuroscience, University of Siena, Policlinico Le Scotte, Viale Bracci 2, 53100 Siena, Italy

**Keywords:** Type 2 diabetes, Bone mineral density, Radiofrequency echographic multi spectrometry (REMS), Osteoporosis

## Abstract

**Background:**

Patients with type 2 diabetes (T2DM) have an increased or normal BMD; however fragility fractures represent one of the most important complications of T2DM.

**Aims:**

This study aimed to evaluate whether the use of the Radiofrequency Echographic multi spectrometry (REMS) technique may improve the identification of osteoporosis in T2DM patients.

**Methods:**

In a cohort of 90 consecutive postmenopausal elderly (70.5 ± 7.6 years) women with T2DM and in 90 healthy controls we measured BMD at the lumbar spine (LS-BMD), at femoral neck (FN-BMD) and total hip (TH-BMD) using a dual-energy X-ray absorptiometry device; moreover, REMS scans were also carried out at the same axial sites.

**Results:**

DXA measurements were all higher in T2DM than in non-T2DM women; instead, all REMS measurements were lower in T2DM than in non T2DM women. Moreover, the percentage of T2DM women classified as “osteoporotic”, on the basis of BMD by REMS was markedly higher with respect to those classified by DXA (47.0% vs 28.0%, respectively). On the contrary, the percentage of T2DM women classified as osteopenic or normal by DXA was higher with respect to that by REMS (48.8% and 23.2% vs 38.6% and 14.5%, respectively). T2DM women with fragility fractures presented lower values of both BMD-LS by DXA and BMD-LS by REMS with respect to those without fractures; however, the difference was significant only for BMD-LS by REMS (*p* < 0.05).

**Conclusions:**

Our data suggest that REMS technology may represent a useful approach to enhance the diagnosis of osteoporosis in patients with T2DM.

## Introduction

The prevalence of type 2 diabetes mellitus (T2DM) is increasing worldwide, especially as a result of our aging society, sedentary lifestyle and the obesity epidemic. Therefore, diabetes and its complications represent a major cause of morbidity and mortality and result in increased economic burden [1].

Besides the well-known renal and cardiovascular complications, the increased risk for fragility fractures has recently been recognized as an important complication of T2DM. In fact, over the past 2 decades several studies showed that T2DM patients presented an increased risk of fragility fractures with respect to individuals without diabetes, although, paradoxically, the bone mineral density (BMD) in those with T2DM is higher than in non-diabetic subjects [2–6].

Several meta-analyses have reported that T2DM patients not only have a 1.5 to threefold higher fracture risk, particularly for hip fractures, but also for all non-vertebral, humerus, wrist and ankle fractures while the evidence for vertebral fractures was lower [7–9]. However, several studies have reported that T2DM patients have a higher risk of vertebral fractures and that this risk is particularly elevated in T2DM postmenopausal women [5, 8, 10, 11]. Moreover, several studies have reported that for a given BMD T-score the fracture risk is higher in T2DM patients with respect to non-diabetic subjects, so suggesting that qualitative bone alterations may play an important role in bone fragility in T2DM [3, 9, 10, 12]. Therefore, both the evaluation of BMD by dual-energy X-ray absorptiometry (DXA) and the common fracture risk assessment algorithms (such as FRAX) underestimate fracture risk in T2DM patients [4, 5, 9, 13]. At present, considering the inadequate reliability of BMD in a T2DM population, there is a growing interest in other techniques complementary to DXA which can improve our ability to determine bone strength and fracture risk in these patients [14]. Among these latter we can include trabecular bone score (TBS), quantitative computed tomography (QCT), microindentation, bone turnover markers and quantitative ultrasound [14, 15]. Recently, an innovative non-ionizing technology, called Radiofrequency echographic multi spectrometry (REMS), has been introduced [15–17]. The operating principle of REMS is based on the analysis of native raw unfiltered ultrasound signals, the so called radiofrequency ultrasound signals, acquired during an echographic scan of lumbar vertebrae and/or proximal femur. The analysis of native unfiltered ultrasound signals allows to retain the maximum information about the characteristics of the investigated tissues, which are normally filtered out during the conventional process of B-mode image reconstruction. The bone health status is assessed through the comparison of the analysed signal spectra with previously derived reference spectral models for the considered pathological and normal conditions. The precision and diagnostic accuracy of REMS as compared to DXA have been already validated [15–17]. Moreover, several recent studies have reported that REMS T-score is able to predict the occurrence of incident fragility fractures in women, representing a promising approach to enhance osteoporosis diagnosis [17, 18].

The aim of this study was evaluate whether the use of the REMS technique may improve the identification of osteoporosis status in T2DM patients.

## Patients and methods

A cohort of 90 consecutive Caucasian women with T2DM referred to the Diabetes Unit of the Department of Internal Medicine at the University Hospital of Siena, between May 2020 and December 2020, were enrolled in the study.

Inclusion criteria were as follows: age between 50 and 80 years, postmenopausal status, body mass index (BMI) between 18.5 and 39.9 kg/m^2^, age at T2DM diagnosis > 30 years, and glycosylated hemoglobin (HbA1c) < 8.5%. The T2DM patients previously treated with antiosteoporosis drugs, except calcium and vitamin D supplements and those who were suffering illness (cancer, multiple myeloma, hyperparathyroidism etc.) or were receiving therapies able to influence bone metabolism (glitazones, glucocorticoids, anticonvulsants etc.), were excluded. All patients had normal serum creatinine levels and no major comorbidities impairing normal daily activity.

The control group included 90 consecutive postmenopausal women, aged 50–80 years, BMI range 18.5–39.9 kg/m^2^ and without T2DM, referred to the Outpatient Clinic of our Department, between May 2020 and December 2020. The non-diabetic patients affected by diseases or treated with drugs known to interfere with bone metabolism were excluded from the study. For all subjects, a detailed personal and familiar medical history was obtained to assess smoking habits, alcohol intake, years since menopause, T2DM duration and other comorbidities. The daily dietary calcium intake was assessed by a validated food frequency questionnaire including foods which account for the majority of calcium in the Italian diet [19]. In addition, height and weight were measured in a standardized fashion and BMI was calculated as weight in kilograms divided by the square of height in meters.

In all subjects we measured BMD at the lumbar spine (LS-BMD), at femoral neck (FN-BMD) and total hip (TH-BMD) using a dual-energy X-ray absorptiometry device (Discovery W, Hologic, Waltham, MA, USA). All DXA scans were performed according to the standard clinical routine procedures. Osteoporosis and osteopenia were diagnosed according to the World Health Organization (WHO) definition: a *T* value lower than − 2.5 was diagnosed as osteoporosis and a *T* value less than − 1.0 but higher than − 2.5 was diagnosed as osteopenia; sex-matched Italian reference data were used for the calculation of T-score.

REMS scans were performed employing a dedicated echographic device (EchoStation, Echolight Spa, Lecce, Italy), equipped with a convex transducer operating at the nominal frequency of 3.5 MHz and used as recommended by the manufacturer. In a REMS investigation, the probe is placed on the abdomen or on the hip in order to visualize of the target bone interface and the operator has to set the appropriate values of scan depth and transducer focus. Subsequently, the software detects the sought bone interfaces in the sequence of acquired frames and identifies the regions of interest for the diagnostic evaluation. The analysis of single scan line spectra allows the automatic exclusion of signals corresponding to artefacts, such as calcifications or osteophytes, thanks to the identification of unexpected spectral features. The selected measured data are finally synthetized in a patient-specific spectrum of the considered bone target, which undergoes an advanced comparison with gender-, age-, site- and BMI-matched reference spectral models extracted from a dedicated database. Actually, the spectral modifications introduced by the physical properties of the bone structure that has backscattered the ultrasound signals are identified by the comparison procedure, resulting in a BMD estimation and in the consequent diagnostic classification as healthy, osteopenic or osteoporotic. Data processing methodologies implemented in the REMS approach were detailed in previous papers [16, 17].

In all subjects, fasting venous blood samples were drawn at baseline in order to assess serum levels of fasting plasma glucose (FPG), glycosylated hemoglobin (HbA1c), 25-hydroxyvitamin D (25OHD), parathyroid hormone (PTH), serum calcium, serum phosphate and creatinine. Serum 25OHD was determined by a chemiluminescence immunoassay (LIAISON 25OHD Total Assay, DiaSorin Inc, Stillwater, MN, USA). In our institution, the intra- and inter-assay coefficients of variation were 6.8% and 9.2%, respectively. Serum PTH was assessed by immunoradiometric assay (Total Intact PTH, Antibodies Lab. Inc.; Santee, CA, USA) and the intra- and inter-assay coefficients of variation were 3.6% and 4.9%, respectively. In T2DM the presence of prior low trauma major fractures (hip, vertebrae, wrist, ankle, humerus) was ascertained by self-report and confirmed by an examination of clinical and radiological reports. Five patients (two diabetic and three non-diabetic) were excluded due to inadequate quality of BMD or REMS measurements. Therefore, the statistical analysis was carried out in 88 diabetic and 87 non-diabetic postmenopausal women.

An informed written consent was obtained from all participants, and the study was approved by the Institutional Review Board of Siena University Hospital (ID-14783/19). All the data were anonymized before being used for the statistical analysis.

### Statistical analysis

All values were expressed as mean ± SD. The Kolmogorov–Smirnov test was used to verify the normality of the distribution of the outcome variables. Clinical data and initial values of the variables measured in the study groups were compared using Student’s *t* test and Mann–Whitney *U* test as appropriate. Categorical variables were compared by Chi-square test or Fisher’s exact test, as appropriate. The associations between different parameters were tested by either Pearson’s correlation or Spearman’s correlation as appropriate or by partial correlation analysis.

All tests were performed using the SPSS statistical package for Windows version 16.0 (SPSS Inc., Chicago).

## Results

The demographic and clinical characteristics of T2DM women and controls are shown in Table 1. There were no significant differences between the two groups for age, height, biochemical parameters, PTH, 25OHD and dietary calcium intake. As expected, BMI was significantly higher (*p* < 0.05) in T2DM than in non-T2DM women. The mean diabetes duration was 14.3 ± 11.3 years. DXA measurements (LS-BMD, FN-BMD, and TH-BMD) were all higher in T2DM than in non-T2DM women, but the differences reached the statistical significance (*p* < 0.01) only for LS-BMD and TH-BMD. Instead, all REMS measurements were lower in T2DM than in non-T2DM women, but the differences did not reach any statistical significance (Table 1). Moreover, ten T2DM women (11.4%) and thirteen non-T2DM women (14.9%) were smokers.

**Table 1:** Demographic and clinical characteristics of the study population

	T2DM patients (*N* = 88)	Controls (*N* = 87)	*p*
Age (years)	70.5 ± 7.6	69.2 ± 7.5	n.s
Weight (kg)	69.2 ± 13.7	66.1 ± 1.3	n.s
Height (cm)	160.0 ± 6.6	160.1 ± 6.8	n.s
BMI (kg/m^2^)	27.0 ± 4.9	25.6 ± 4.0	0.05
Calcium intake /mg/daily)	872.2 ± 280.3	891.2 ± 255.7	n.s
HbA1c (%)	7.0 ± 1.1	–	
T2DM duration (years)	14.3 ± 11.34	–	
Creatinine (mg/dl)	0.9 ± 0.3	0.9 ± 0.2	n.s
Calcium (mg/dl)	9.3 ± 0.6	9.2 ± 0.5	n.s
Phosphate (mg/dl)	3.6 ± 0.5	3.4 ± 0.5	n.s
25OHD (ng/ml)	21.0 ± 9.9	24.4 ± 8.9	n.s
PTH (pg/ml)	36.3 ± 19.7	34.8 ± 17.9	n.s
DXA LS-BMD (g/cm^2^)	0.984 ± 0.180	0.906 ± 0.142	0.01
DXA FN-BMD (g/cm^2^)	0.735 ± 0.131	0.699 ± 0.118	n.s
DXA TH-BMD (g/cm^2^)	0.860 ± 0.123	0.809 ± 0.112	0.01
REMS LS-BMD (g/cm^2^)	0.812 ± 0.106	0.841 ± 0.090	n.s
REMS FN-BMD (g/cm^2^)	0.632 ± 0.120	0.636 ± 0.059	n.s
REMS TH-BMD (g/cm^2^)	0.758 ± 0.137	0.770 ± 0.067	n.s

**Fig. 1:**
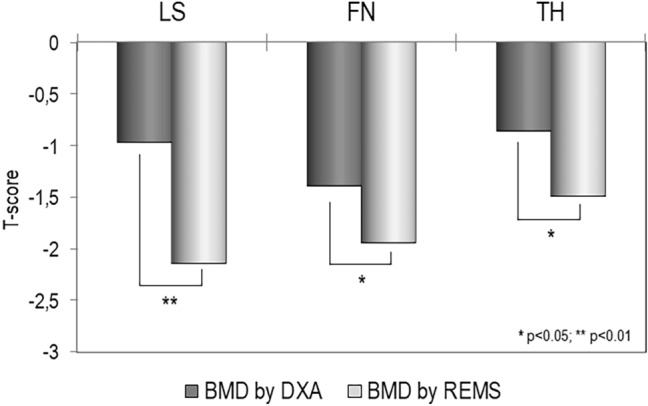
Values of BMD expressed as T-score at lumbar spine (LS), at femoral neck (FN) and at total hip (TH) by DXA and REMS technique in T2DM patients

Figure 1 shows the mean values of BMD at different skeletal sites, expressed as T-score, obtained by DXA and REMS technique. It is evident that BMD T-score by REMS were significantly lower than those obtained by DXA technique both at lumbar spine (*p* < 0.01) and at all femoral sub-regions (*p* < 0.05).

**Fig. 2:**
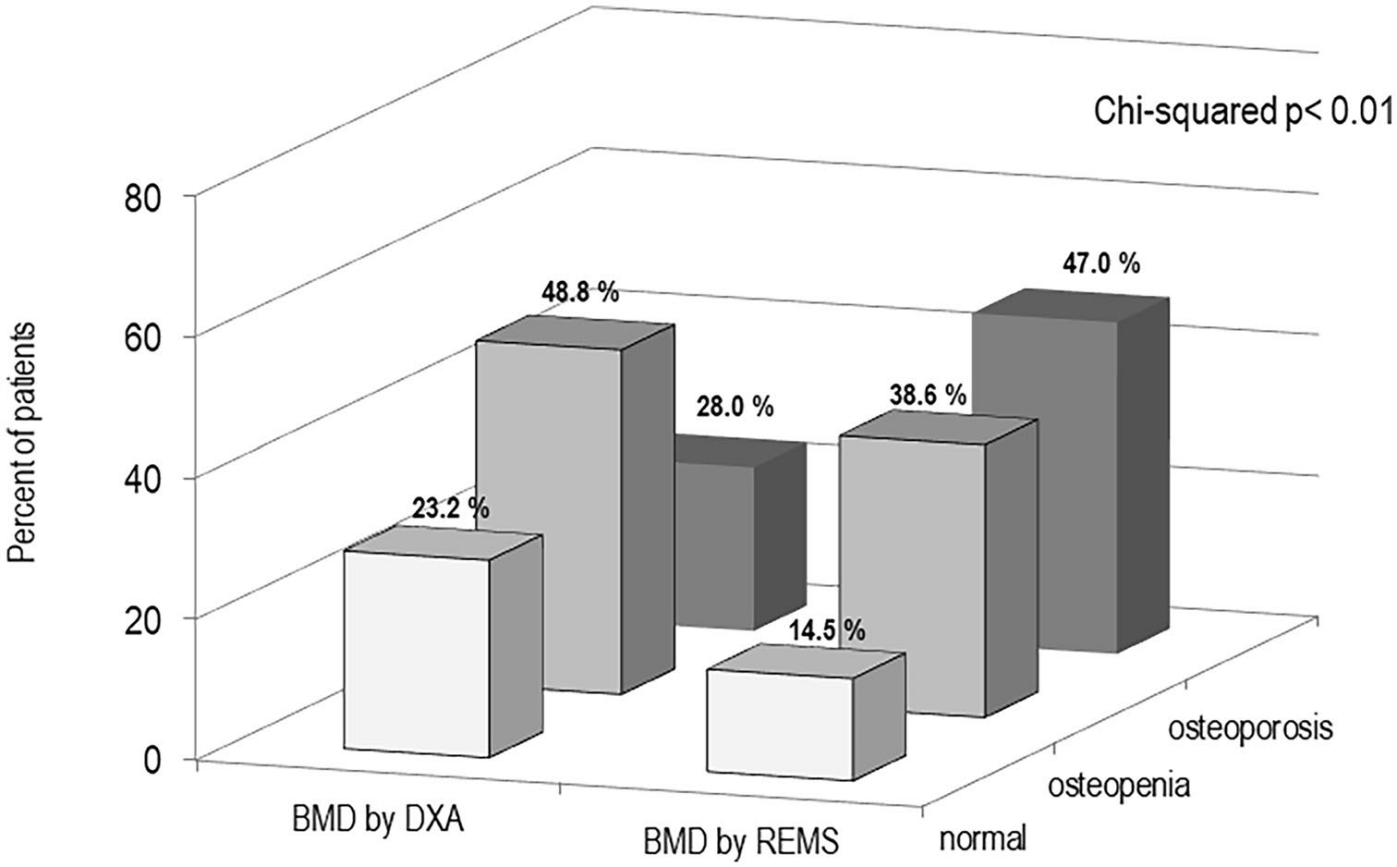
Percentage of T2DM women classified as “osteoporotic”, “osteopenic” or “normal” on the basis of BMD T-score values obtained by DXA and REMS technique

Figure 2 shows the percentage of T2DM women classified as “osteoporotic”, “osteopenic” or “normal” on the basis of BMD T-score values obtained by DXA and REMS technique, respectively. It’s evident that the REMS technique allows a greater number of T2DM patients to be classified as osteoporotic than DXA (47.0% vs 28.0%, respectively). On the contrary, the percentage of T2DM women classified as osteopenic or normal by DXA was higher with respect to that by REMS (48.8% and 23.2% vs 38.6% and 14.5%, respectively).

Table 2 presents the age and BMI adjusted partial correlations of BMD values by DXA and REMS with HbA1c and T2DM duration. The BMD by REMS at all skeletal sites and TH-BMD by DXA were inversely associated with T2DM duration. No significant associations between BMD by REMS and HbA1c levels were observed.

**Table 2:** Age and BMI adjusted partial correlations of BMD values by DXA and REMS technique with HbA1c serum level and T2DM duration

	HbA1c (%)	T2DM duration (years)
DXA BMD-LS (g/cm^2^)	− 0.22*	− 0.09
REMS BMD-LS (g/cm^2^)	− 0.07	− 0.21*
DXA BMD-FN (g/cm^2^)	− 0.24*	0.04
REMS BMD-FN (g/cm^2^)	− 0.04	− 0.24*
DXA BMD-TH (g/cm^2^)	− 0.26*	− 0.27*
REMS BMD-TH (g/cm^2^)	− 0.07	− 0.23*

*p < 0.05

**Fig. 3:**
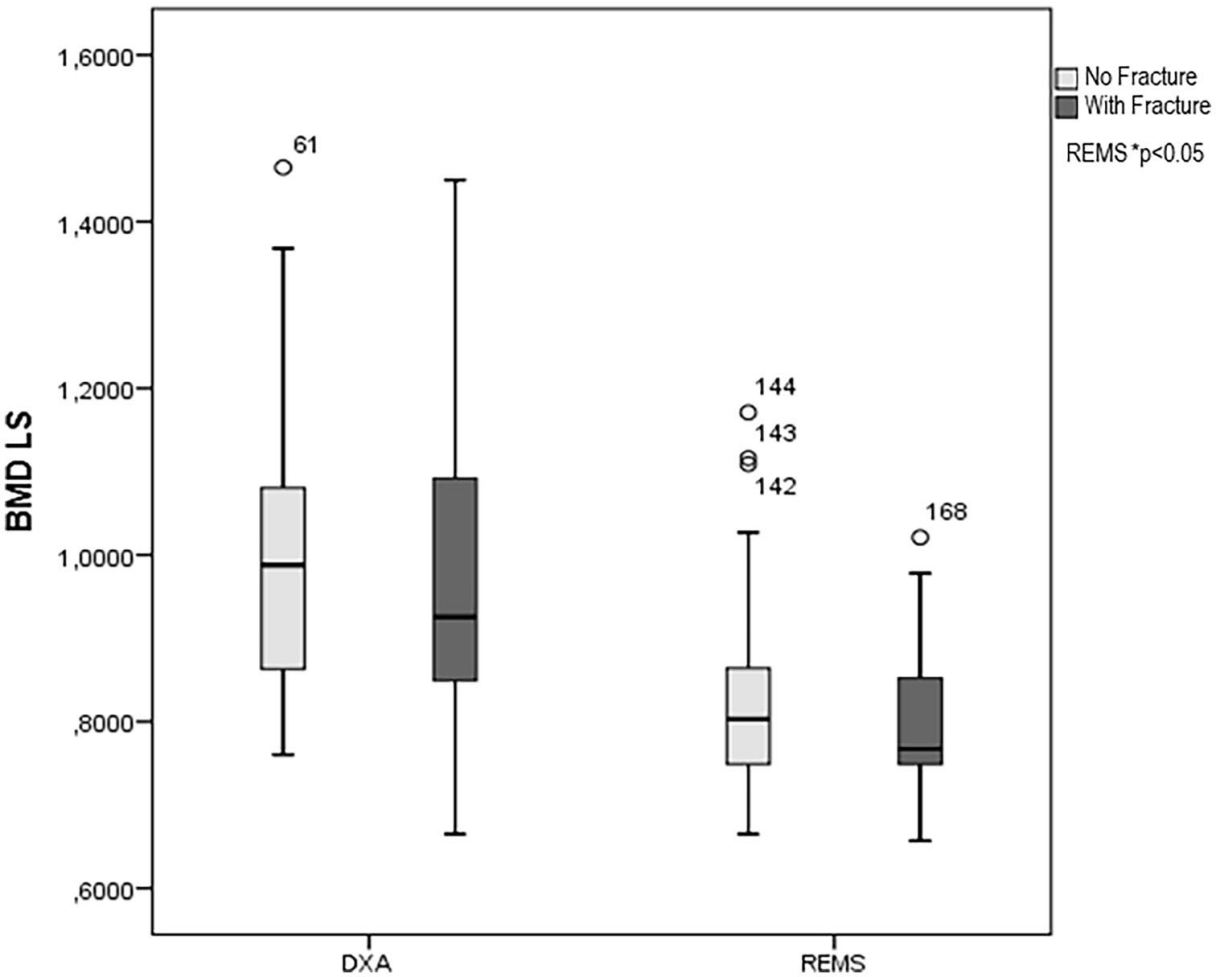
BMD-LS by DXA and REMS technique in DM2 patients with fracture or without fracture

Twenty-two (= 25.0%) T2DM women had a history of low-trauma major fractures. The values of BMD-LS, measured by DXA and REMS technique, in T2DM patients with or without prior low-trauma fractures are shown in Fig. 3. As expected, the T2DM women with previous major fragility fractures presented lower values of both BMD-LS by DXA and BMD-LS by REMS with respect to those without fractures; however, the difference was significant only for BMD-LS by REMS (*p* < 0.05).

## Discussion

To the best of our knowledge, this is the first study aimed to evaluate the usefulness of the REMS technique in determining bone status in postmenopausal women with T2DM. The main finding of this study is represented by the fact that while BMD by DXA values, as expected, were higher in women with T2DM than in controls, REMS-estimated BMD values were lower in women with T2DM than in controls. As a result, BMD by REMS allowed classification of a greater number of T2DM women as “osteoporotic”, compared to BMD by DXA. Therefore, our data suggest that REMS-estimated BMD could be a good diagnostic tool in demonstrating the diabetes-associated bone disease.

With the progressive aging of the population, bone fragility with consequent low trauma fractures is becoming one of the most important complications of diabetes. BMD by DXA, although this remains one important risk factor for fracture in T2DM, is considered to have a poor predictive value for fragility fractures in T2DM patients [20]. In fact, several studies reported that fracture risk is almost twice as high in patients with T2DM compared to subjects without T2DM, even though patients with T2DM have an increased or normal BMD [3, 6, 11, 21]. Therefore, both the evaluation of BMD by DXA and the common fracture risk assessment algorithms (such as FRAX) underestimate fracture risk in T2DM patients [4, 5, 9]. In recent years, several options have been proposed to improve the performance of FRAX in T2DM (including rheumatoid arthritis as a proxy for the effects of DM; reducing the femoral neck T-score by 0.5 SD; increasing the age input to by 10 years; making a TBS adjustment to FRAX) but none of these methods were optimal in predicting the risk of major fragility fractures in these patients [22].

An interesting result of this preliminary study is represented by the fact that T2DM women with previous major fragility fractures presented values BMD-LS by REMS significantly lower with respect to those without fracture; this finding suggest that REMS technique could be better than DXA in assessing fracture risk in T2DM women.

The increased skeletal fragility in T2DM patients in spite of the presence of normal or even increased bone mineral density can be explained by an impaired bone quality with a consequent reduction of bone strength. Unfortunately, nowadays, bone quality can only be adequately assessed with invasive methods which, therefore, are not easily available in clinical practice [5, 12, 13, 15]. Therefore, there is growing interest in identifying new easy-to-use and reliable techniques which can improve our ability in the assessment of bone status and fracture risk in these patients [14]. In particular, the techniques that use quantitative ultrasound appear very attractive both because they measure the bone properties using the attenuation and reflection of pulse ultrasound waves and because they have some advantages over DXA such as low cost, portability and absence of ionizing radiation. Moreover, QUS parameters at calcaneous were reported to be able to predict osteoporotic fractures with similar sensitivity but lower specificity than DXA [23].

However, data concerning the use of QUS parameters in discriminating T2DM patients with or without fragility fractures are scarce and conflicting [12, 24, 25], and a study has reported that calcaneal QUS was no different between T2DM patients with prevalent vertebral fractures compared to those without [26]. On the other hand, the current use of QUS parameters as a clinical diagnostic tool has found significant limitations in the fact that QUS parameters are measurable only on peripheral skeletal sites and that currently too many QUS devices are available each differing from another for measurement techniques and the measured parameters [15]. These limits have been overcome by the introduction of REMS technology. The advantages of REMS technology include the use of non-ionizing radiation, the analysis of axial sites, the high accuracy and reproducibility and the ability to predict the risk of incident fragility fractures [15, 17, 18]. In particular, the results of this exploratory investigation suggest that REMS technology may be useful in the assessment of impaired bone quality in patients with T2DM. In fact, REMS-estimated BMD is low in T2DM patients and inversely associated with DM duration. Therefore, the pattern of BMD by REMS appears similar to that of TBS, a parameter related to the structure of the trabecular bone of the vertebrae, which has been reported to be low in T2DM and more useful than BMD in predicting fracture risk [22].

At present, insufficient data are available to hypothesize how REMS can be affected by qualitative skeletal changes in diabetes mellitus (such as increased cortical porosity, microarchitecture abnormalities, turnover reduction, etc.). However, REMS approach might have the potential to also calculate parameters different from BMD, derived from bone quality indicators and further related to bone strength [5, 15, 27].

Also the fact that REMS was inversely associated with DM duration may be important since several studies found that a T2DM duration longer than 10 years significantly increases fragility fracture risk, regardless of diabetes control [7, 13]. Instead, no significant associations between BMD by REMS and HbA1c were observed. However, it is important to consider that the predictive values of a single HbA1c measurement in the assessment of fracture risk may be questionable [13].

The observed differences between BMD by DXA and BMD by REMS might also be due to the fact that several artifacts on the DXA imaging can affect the scan results [28]. In fact, degenerative changes due to osteoarthritis, osteophytes, and vascular calcifications or vertebral fractures will produce a false overestimation of effective BMD values and consequently a significant underestimation of fracture risk [28]. In particular, idiopathic skeletal hyperostosis is common among patients with diabetes mellitus [5]. Moreover, recently Veronese et al. reported that T2DM is linked to osteoarthritis outside of excess weight and that T2DM may play a role in osteoarthritis pathophysiology [29].

REMS technology by the analysis of native raw unfiltered ultrasound signals appears to be able to recognize and automatically remove the raw signals from calcifications, osteophytes and other possible causes of artifacts and overcome the most common artifacts that affect the value of the BMD by DXA, so making the correct definition of the bone status [30].

Our study has some limitations. First, the cross-sectional nature of the study does not allow the establishing of any causality relationships between the parameters. Second, the fact that HbA1c was measured only at the time of study does not permit excluding that glycemic control was different in the months/years before enrolment. Third, the study was carried out on a cohort of elderly women with long-lasting diabetes; therefore, the results may not be reproducible in diabetic populations with different characteristics.

In conclusion, the results of this exploratory investigation suggest that REMS technology may represent a useful approach to enhance the diagnosis of osteoporosis in patients with T2DM. Further studies are warranted in order to confirm these preliminary data and to establish new REMS based parameters related to bone quality which may improve the prediction of fracture risk in diabetic patients.
